# Mapping the Quality of German-Language Health Information on the Treatment of Knee Osteoarthritis: Cross-Sectional Analysis

**DOI:** 10.2196/78007

**Published:** 2025-12-11

**Authors:** Sandro Zacher, Jürgen Kasper, Julia Lauberger, Julia Lühnen, Lisa-Marie Redlich, Anke Steckelberg

**Affiliations:** 1 Institute of Health, Midwifery and Nursing Science Medical Faculty of Martin Luther University Halle-Wittenberg, University Medicine Halle Martin Luther University Halle-Wittenberg Halle (Saale) Germany; 2 Department of Nursing and Health Promotion Faculty of Health Sciences OsloMet – Oslo Metropolitan University Oslo Norway; 3 Institute of Clinical Nursing Science Charité - Universitätsmedizin Berlin, corporate member of Freie Universität Berlin and Humboldt-Universität zu Berlin Berlin Germany

**Keywords:** consumer health information, evidence-based health information, health literacy, informed choice, knee osteoarthritis, quality, total knee replacement

## Abstract

**Background:**

Patients with knee osteoarthritis have a considerable need for information about their condition, its progression, and available treatments. Decision-making is often complex and requires evidence-based health information material (HIM). When medical consultations do not sufficiently address patients’ needs, many seek additional information independently.

**Objective:**

This study aimed to examine the quality of German-language HIM on knee osteoarthritis treatment and its suitability for supporting informed choice. In particular, the study analyzed the content of the HIM and assessed the balance in the presentation of treatment options.

**Methods:**

A descriptive cross-sectional study was conducted. HIM was identified through a combination of search strategies, including a systematic internet search using commonly used German terms related to the treatment of knee osteoarthritis. Identified HIMs were independently assessed by 2 raters using the validated Mapping the Quality of Health Information (MAPPinfo) checklist, which operationalizes the criteria of the Guideline Evidence-Based Health Information. Information quality was calculated on a scale from 0% to 100%, representing compliance with the quality standard. A descriptive content analysis was also carried out to examine the range and balance of treatment options presented, as well as the reporting of benefits and complications associated with total knee arthroplasty (TKA). The presence of certification was recorded.

**Results:**

A total of 94 HIMs were included. On average, the material met 14.6% (SD 9.4%) of the quality criteria. HIM from public and nonprofit providers performed better (mean 40.1%, SD 3.6% and mean 37.2%, SD 23.1%, respectively) than those from other providers. Overall, 14 HIMs presented treatment options in a balanced manner. Among the 78 HIMs that covered TKA, 38.5% (n=30) did not report any benefits, and 35.9% (n=28) omitted potential complications. Certified HIMs showed only moderately higher information quality than uncertified material (mean 26.8%, SD 16% vs mean 12.7%, SD 5.9%).

**Conclusions:**

Our results highlight the urgent need to improve the quality of German-language HIM on knee osteoarthritis. The deficits identified are fundamental and affect all dimensions of information quality. Although HIM from public or nonprofit organizations has better information quality, this does not facilitate informed choice. The frequent omission of complications and benefits of TKA and the unbalanced presentation of treatment options can influence decisions. Until structural improvements are made, patients seeking quality information should favor material from public or nonprofit providers. Additionally, the MAPPinfo checklist could form the basis of a differentiated certification system to make information quality more transparent for patients.

## Introduction

Knee osteoarthritis is a globally prevalent, chronic degenerative joint disease that poses a significant burden on affected individuals, particularly in advanced stages. It is characterized by progressive cartilage degradation but affects the entire knee joint, leading to pain, stiffness, and restricted mobility. These factors significantly impair patients’ quality of life and mobility [1]. In 2020, the self-reported 12-month prevalence of osteoarthritis among adults aged 18 years and over in Germany was 17.1%. The condition affected women more frequently than men, with prevalence rates of 21.6% and 12.4%, respectively. The prevalence increased substantially with age [2]. Another study estimated the all-time age- and gender-standardized relative prevalence of knee osteoarthritis at 10.6% (11.8% in women and 9.4% in men) [3].

Treatment options range from conservative interventions such as physiotherapy and pain management to surgical procedures, including joint-preserving and joint-replacing interventions [1,4]. Total knee arthroplasty (TKA) is an established treatment option associated with high expectations regarding pain relief and the restoration of mobility [5]. In 2019, Germany, along with Switzerland, Finland, and Austria, had the highest rates of TKA. While the Organisation for Economic Co-operation and Development average was 137 procedures per 100,000 inhabitants, these countries performed over 200 TKA [6]. A German study on second opinions found that only 40% of recommendations in favor of TKA procedures were confirmed by a second orthopedic surgeon in an arthroplasty center [7]. Studies on patient satisfaction following TKA show heterogeneous results, influenced by methodological limitations related to measurement tools and study quality [8,9]. Dissatisfaction can stem from unmet expectations, which in turn may result from insufficient or unbalanced provision of information [5].

The decision for or against a particular treatment option for knee osteoarthritis is complex [10]. Insufficient or inadequate information can be a major barrier to informed decision-making [11]. Various studies suggest that patients feel inadequately informed about their condition, its prognosis, and the available treatment options [11-13]. Moreover, TKA is frequently perceived as an inevitable treatment choice [10,12,14,15]. Research indicates that conservative treatment options are often not offered, used, or fully explored before surgical interventions take place [12,13,15,16]. At the same time, there is a clear patient demand for comprehensive information on conservative treatment options, including their benefits and risks [17,18]. The provision of health information material (HIM) regarding TKA can be challenging for various reasons. Decisions in favor of surgery are sometimes made before a formal indication has been established, leading to selective information dissemination [12]. Furthermore, information provided during the indication process for TKA tends to emphasize the benefits of surgery, and patients generally tend to overestimate the benefits of such a procedure while underestimating its risks [12,19].

Dissatisfaction with information provision can lead patients to seek information independently, particularly online [20]. Individuals with chronic illness are among the most frequent users of online health information [21]. Internet search behavior is influenced by factors such as age, gender, socioeconomic status, and education level [20,22]. Despite the importance of doctors and family members as primary sources of information, many individuals rely on the internet as a key research platform for health-related topics [23,24]. Depending on the study, between 68% and 74% of German respondents report using the internet to search for HIM [24,25]. The most commonly used sources include specialized health websites, websites of doctors, hospitals, and health care institutions, as well as commercial websites. Information on treatment options is most frequently sought on the websites of doctors and hospitals [24]. Trust in online HIM varies considerably and is often influenced less by content itself than by factors such as readability and design [21,23].

Informed choice is increasingly recognized as a quality standard in health care and is embedded in ethical principles [26]. An informed choice is defined as one based on the best available evidence regarding the benefits and risks of all treatment options and aligned with the patient’s personal preferences [27]. With the introduction of the German Patients’ Rights Act in 2013, which was incorporated into the German Civil Code (Section 630a-630h BGB), comprehensive patient information became a legal requirement. Furthermore, the inclusion of TKA in the second opinion process in 2021 reinforced the importance of an informed choice [28]. A key requirement for an informed choice is the availability of evidence-based HIM, which objectively, transparently, comprehensively, and understandably presents the advantages and disadvantages of medical or health care interventions. The 2017 Guideline Evidence-Based Health Information (Guideline EBHI) provided recommendations for the development of such material [29].

The quality of HIM with regard to knee osteoarthritis and TKA has already been assessed with several tools and in various countries. Findings indicate that quality varies widely and is often deemed inadequate [30-36]. None of the tools used in the previously conducted studies is considered fully adequate to comprehensively assess the quality of HIM based on the quality concept of the Guideline EBHI [37].

This study aimed to evaluate the quality of German-language HIM on the treatment of knee osteoarthritis. Specifically, it sought to determine whether German-speaking patients can make an informed choice based on the available HIMs. In particular, the study analyzed the content of HIM, assessed the balance in the presentation of treatment options, and identified key characteristics of the HIM.

## Methods

### Design

This study is part of a project to develop an evidence-based informed consent form for treatment decisions related to knee osteoarthritis [38]. Using a descriptive cross-sectional design, this study assesses compliance with evidence-based quality standards in HIM about knee osteoarthritis treatment. Initially, the plan was to evaluate the quality of printed information leaflets distributed by doctors and received by patients enrolled in an exploratory study [12]. However, due to the low response rate to the leaflet collection, the methodology was adapted to include online HIM. The Strengthening the Reporting of Observational Studies in Epidemiology (STROBE) statement was used for the reporting [39].

### Sample and Search Strategy

From January to July 2021, we invited doctors and patients participating in an exploratory study on information and decision-making processes to provide printed material on the treatment of knee osteoarthritis [12]. Specifically, we asked 3 general practitioners, 4 office-based orthopedic specialists, and 1 hospital in an urban metropolitan area in western Germany to contribute. In addition, 13 patients, recruited both regionally and nationwide, were invited to contribute material. Participants were asked to send their material to us electronically; for those unable to do so, prepaid return envelopes were provided. However, only sporadic printed material was received from doctors, and none from patients. To supplement the collected data, we conducted an exploratory internet search in April 2022 using the Google search engine. This search targeted German-language information provided specifically by medical practices and clinics. Using the German term for knee osteoarthritis, the first 5 pages of search results were reviewed, with browser settings reset to default. This first step aimed to provide an overview of the type and scope of health information available.

In May 2023, a systematic search was conducted to expand the scope of the existing data. This search was also performed using Google, the most widely used search engine in Germany [40], with browser settings reset and incognito mode activated to minimize personalization of search results. We used Google Trends analysis [41] to identify the most popular German search terms relating to treatments for knee osteoarthritis. As TKA was a popular topic, a separate search term was used for this treatment option. For knee osteoarthritis, the term “Kniearthrose” was used, while for TKA, the term “Knie TEP” was applied. The initial 50 search results for each search term were extracted and documented in a Microsoft Excel spreadsheet. Additionally, known sources of health information not captured in the Google search results were manually added to ensure comprehensiveness.

### Eligibility Criteria and Screening

HIM was included if it met the following criteria: (1) it provided information about the treatment of knee osteoarthritis, (2) addressed lay audiences, and (3) was written in the German language.

For the internet search, additional criteria were applied, such as websites were included regardless of how the content was presented. Google results marked as advertisements were excluded. Websites were also excluded if (1) they consisted exclusively of patient testimonials or (2) the information was not publicly accessible.

HIM identified across all 3 searches was consolidated, and duplicates were removed. When multiple websites from the same provider addressed different search terms, they were counted as a single HIM if the content was clearly interconnected and there was overall coherence. We defined the coherence of HIM on the basis of the structure. We consider a system of hierarchically organized pages from the same provider to be one HIM, whereas a referral to another provider, for example, is not considered to be part of the same HIM.

Selection criteria were independently reviewed by 2 researchers, and disagreements were resolved by a third reviewer. For the internet searches, copies of the included and excluded HIM were saved as PDF files so that the original could be accessed in the event of website changes during the course of the study.

### Data Collection

All HIMs were categorized based on 3 basic variables: provider, country, and certification. Providers were classified into 8 categories, including commercial providers, hospitals, medical practices, nonprofit organizations, publicly funded providers, specialist associations, health insurance companies, and self-help groups. As German-language HIM can come from various countries, a further classification was made according to the provider’s country of origin, that being Germany, Austria, or Switzerland. Regarding certification, distinctions were made based on whether the HIM held any external certification or recommendation and, if so, from which organization.

### Quality Assessment

The quality assessment was carried out using the Mapping the Quality of Health Information (MAPPinfo) checklist [42], which is based on the Guideline EBHI [29]. It is the first tool to implement the recommendations of the guideline in a precise way, allows the assessment to be based only on HIM without the need for the search of additional information, and can be reliably used by people without special training or expertise in evidence-based practice. Therefore, the quality assessment process is much more transparent and accessible to a wider audience. The MAPPinfo checklist consists of 19 items that evaluate the following 4 key aspects of quality: definitions, which refer to the target audience and the purpose of the information; transparency, which addresses how the information was prepared, including details on authorship, funding, timeliness, and sources; content, which focuses on the informational content such as the explanation of options and the presentation of benefits and harms; and presentation, which examines the suitability of how the content is delivered. The checklist includes 11 items assessed using a dichotomous format (fulfilled/not fulfilled) and 8 items assessed using a trichotomous format (fulfilled/partially fulfilled/not fulfilled). The total number of applicable items depends on the specific content of the HIM, with certain items, such as C7/P4, used only for diagnostic decisions and others, such as P7, applied only when charts are used to visualize frequencies. The total score of the MAPPinfo checklist, which ranges from 0% to 100%, represents the information quality and reflects the extent to which the criteria of the Guideline EBHI are met.

The psychometric properties of the checklist had been validated in previous studies, confirming its suitability [42]. The assessment process and documentation were carried out using the Microsoft Excel–based MAPPinfo Evaluation Toolkit, which includes several worksheets. These enabled the collection of independent evaluator ratings, plausibility checks, identification of discrepancies, and documentation of consensus, as well as tables with calculations of reliability and information quality and the visualization of the results [43].

The MAPPinfo rating was carried out independently by 2 master students (LMR and JS), followed by a consensus process. In the case of disagreement, a third rater (SZ) was consulted, and consensus was reached through discussion. To ensure high interrater agreement, the 3 raters independently assessed a sample of 10 preselected HIMs and discussed discrepancies in their assessments, developing a common understanding of how the criteria should be applied to the specific health problem of knee osteoarthritis.

### Content Mapping

The MAPPinfo checklist already includes the aspects of trustworthiness and appropriate presentation of content. In addition, the content of the HIM was systematically extracted using predefined criteria, including information on the complications and benefits of TKA, treatment options for knee osteoarthritis, anesthesia options, and complications related to anesthetics. A subjective evaluation was also conducted to assess whether the treatment options were presented in a balanced manner. The criteria for complications of TKA were determined using the standardized list of the Knee Society [44], the criteria for benefits of TKA and the treatment options were defined using the developed evidence-based informed consent forms [38], and the definitions of the German consensus guideline on knee osteoarthritis [4]. The extraction process was organized in an Excel matrix, with both the criteria and the matrix itself piloted and refined based on an initial review of the 10 preselected HIMs. Where classification was not feasible, free-text entries were permitted to capture relevant details. The assessment was initially conducted by one rater and subsequently reviewed by a second rater to ensure consistency and accuracy.

### Data Analyses

Data were analyzed using the Excel-based MAPPinfo Evaluation Toolkit [43]. We calculated a score ranging from 0% to 100% for each HIM. Each item was assigned 1 point if it was fulfilled, 0.5 points if it was partially fulfilled, or 0 points if it was not fulfilled. Items C4/P1 to C7/P4, which reflect both content and presentation, were given double weighting. The total score was calculated by dividing the sum of all item points by the maximum possible points for all applicable items and expressing the result as a percentage. A score of 100% indicates full compliance with the quality criteria of the Guideline EBHI. Additionally, we determined the overall mean score for all HIMs, as well as the mean scores for each item, including the SD. The overall mean values were further analyzed descriptively for different values of the basic variables of the HIM. The content analysis was performed descriptively, using frequencies and proportions. Furthermore, a descriptive analysis was conducted to evaluate the presentation of treatment options by provider groups. The interrater reliabilities of the MAPPinfo ratings between the 2 raters were calculated at the item level using the T-coefficient. This method does not estimate the probabilities of expected agreement empirically from the given dataset but uses theoretical assumptions derived from the number of potential values that an item can receive [45]. Interrater reliabilities were considered moderate between 0.40 and 0.60, strong when higher than 0.60, and excellent when higher than 0.80 [46].

### Ethical Considerations

As this study does not involve participants or participant data, ethical approval and informed consent were not applicable.

## Results

### Overview

**Figure 1:**
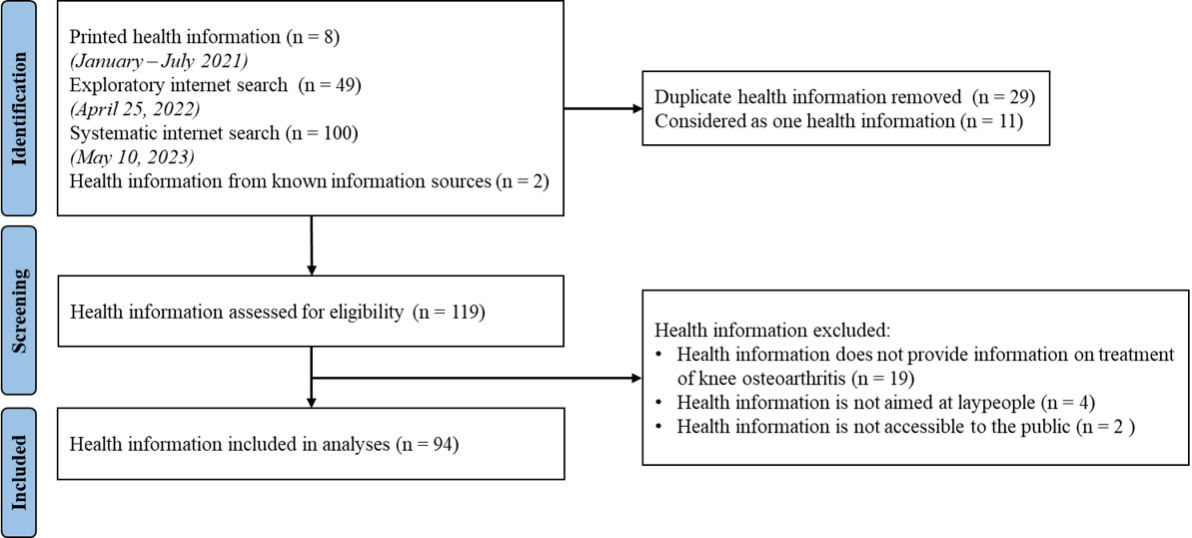
Flowchart of the search and selection of health information material.

Out of 159 identified HIMs, a total of 94 were included in the analysis (Figure 1). Within the total sample, commercial providers accounted for 37.2% (n=35), hospitals for 27.7% (n=26), medical practices for 25.5% (n=24), nonprofit organizations and publicly funded providers for 3.2% (n=3), professional associations for 2.1% (n=2), and health insurance companies for 1.1% (n=1). No HIMs from self-help groups were included. Certifications from 3 organizations were identified (Health Information System Action Forum [Aktionsforum Gesundheitsinformationssystem] (Afgis) [47], German Health Literacy Network [Deutsches Netzwerk Gesundheitskompetenz] (DNGK) [48], and Public Health Foundation [49]), most of which contain transparency criteria. In total, 13 of the analyzed HIMs were certified by at least one of these organizations, while 81 had no certification.

### MAPPinfo Quality Assessment

The agreement between the raters was excellent (T=0.91, T minimum=0.6, T maximum=1). The 94 HIMs demonstrated a mean compliance of 14.6% (SD 9.4%) with the criteria outlined in the Guideline EBHI. Compliance with the quality standards ranged from 0% to 69% among individual HIMs. None of the HIMs met all criteria.

**Figure 2:**
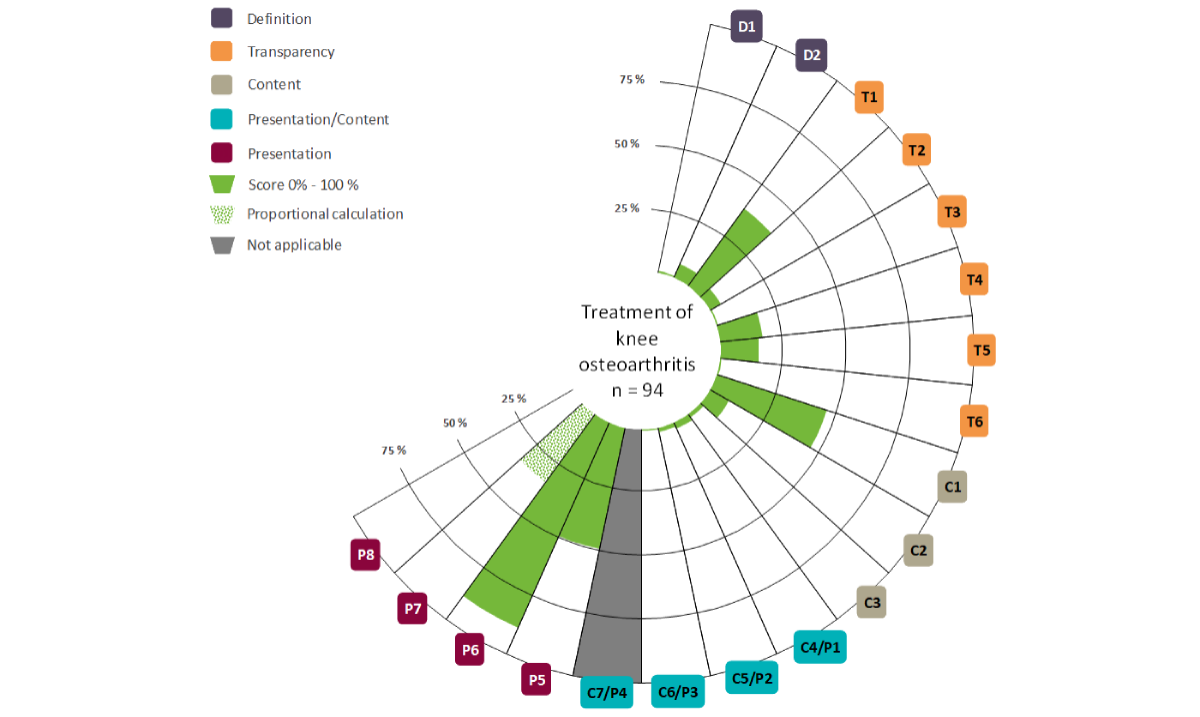
Visualization of information quality created with the Mapping the Quality of Health Information Evaluation Toolkit. P7 only applies to 6 out of 94 health information materials and was therefore calculated proportionally.

At the item level (Figure 2 [43]), the 3 criteria with the highest compliance were the avoidance of using narratives (P6; mean 88.3%, SD 32.1%), the use of neutral language (P5; mean 48.9%, SD 50%), and the explanation of the health problem (C1; mean 45.7%, SD 49.8%). Compliance with the quality standards (Table 1) was very low in some of the criteria. These include, for example, the use of gain and loss framing (P8; 0%), the reporting of a systematic search strategy (T6; mean 1.1%, SD 10.3%), the reporting of a strategy for managing conflicts of interest (T3; mean 1.1%, SD 10.3%), and the presentation of benefits (C5/P2; mean 2.1%, SD 14.4%) and harms (C6/P3; mean 1.1%, SD 10.3%).

Different levels of information quality were observed between provider groups, as well as between HIMs with or without certification, and between HIMs with different reporting of treatment options. HIM provided by publicly funded providers (n=3) and nonprofit organizations (n=3) achieved higher information quality with an average of 40.1% (SD 3.6%) and 37.2% (SD 23.1%), respectively, compared to HIM from other providers (Table 2). HIMs with at least 1 certification (n=13) demonstrated higher information quality than uncertified HIMs (n=81), with an average of 26.8% (SD 16%) compared to 12.7% (SD 5.9%). However, information quality also varied within certified HIMs. Those provided by organizations recognized as reliable health portals by the DNGK [48] had higher information quality than other certifications. HIM providing balanced information on conservative and surgical treatment options (n=14) achieved higher information quality than those focusing on surgical (n=52) or conservative options (n=28). Information quality was comparable across countries of origin (Table 2).

**Table 1:** Information quality according to criteria.

Category	Criterion	Compliance (%), mean (SD)
D^a^1	The target group addressed by the HIM^b^ is clearly defined.	1.1 (10.3)
D2	The HIM explains explicitly that an informed choice about a concrete problem should be facilitated.	6.4 (20.9)
T^c^1	The authors of the HIM are named.	37.8 (44.2)
T2	The funding source of the HIM is disclosed.	5.3 (22.4)
T3	A strategy for managing conflicts of interest is disclosed.	1.1 (10.3)
T4	The HIM indicates how up-to-date it is.	17 (26.8)
T5	The sources of information are named.	15.4 (28.3)
T6	The systematic search strategies underlying the generation of information are transparent.	1.1 (10.3)
C^d^1	The health problem is explained.	45.7 (49.8)
C2	The options are named and explained.	9 (20.6)
C3	The HIM makes statements about stochastic uncertainty.	2.1 (14.4)
C4/P^e^1	The natural course (in the case of diagnostic problems: the prevalence) of the disease is adequately presented.	3.2 (17.6)
C5/P2	The benefits are presented adequately.	2.1 (14.4)
C6/P3	The harms are presented adequately.	1.1 (10.3)
C7/P4	For diagnostic problems, information on the quality of the test is presented adequately.	N/A^f^
P5	The HIM uses neutral language throughout.	48.9 (50)
P6	The HIM does not use narratives that present relevant factual information.	88.3 (32.1)
P7	Graphics are designed in a suitable manner.	33.3 (37.3)^g^
P8	Information about benefits and harms is supplemented with complementary information (gain and loss framing).	0 (0)

^a^D: definition.

^b^HIM: health information material.

^c^T: transparency.

^d^C: content.

^e^P: presentation.

^f^Not applicable.

^g^Proportionally calculated for 6 out of 94 health information materials.

**Table 2:** Information quality according to the basic variable of the health information materials (HIMs).

Characteristics of health information			Number of HIMs		Information quality (%)		
					Mean (SD)		Minimum-maximum
**Provider**							
	Commercial provider	35		14.3 (7.2)		0-30	
	Hospital	26		11.4 (4.2)		0-20	
	Medical practice	24		12.6 (4.7)		2.5-22.5	
	Nonprofit organization	3		37.2 (23.1)		15-69	
	Publicly funded provider	3		40.1 (3.6)		35-42.9	
	Professional association	2		13.8 (1.25)		12.5-15	
	Health insurance provider	1		7.5 (0)		N/A^a^	
**Origin**							
	Germany	82		14.6 (9.9)		0-69	
	Switzerland	7		15.7 (4.2)		10-22.5	
	Austria	5		13.5 (6.6)		5-25	
**Certification (multiple selections possible)**							
	No certification	81		12.7 (5.9)		0-30	
	At least 1 certification	13		26.8 (16)		10-69	
	Afgis^b^	9		26.5 (16.3)		12.5-69	
	DNGK^c^	5		40.9 (17.3)		15-69	
	Public Health Foundation	1		15 (0)		N/A	
**Presentation of treatment options**							
	Balanced	14		24.2 (17.3)		5-69	
	Surgery-focused	52		12.6 (5.1)		0-25	
	Conservative treatment–focused	28		13.5 (6.8)		0-30	

^a^N/A: not applicable.

^b^Afgis: Health Information System Action Forum [Aktionsforum Gesundheitsinformationssystem].

^c^DNGK: German Health Literacy Network [Deutsches Netzwerk Gesundheitskompetenz].

### Content Analyses

TKA was mentioned as a treatment option by 78 of the 94 HIMs. Within this subgroup, a third of the HIM did not report any complications (n=28, 35.9%) or benefits (n=30, 38.5%). The most commonly reported complications were joint infection (n=39, 50%), prosthesis loosening (n=30, 38.5%), and revision (n=25, 32.1%). In terms of benefits, pain relief was the most commonly reported benefit (n=32, 41%), followed by nonspecific statements about mobility (n=24, 30.8%) and general nonspecific statements about benefits (n=20, 25.6%; Table 3).

Within the same subgroup, 21 (26.9%) HIMs addressed the topic of anesthetics. When anesthetics were mentioned, the available options (different types of anesthetics) were mostly described. A total of 2 (9.5%) HIMs reported associated complications (Table 3).

The HIMs mentioned a range of treatment options for knee osteoarthritis. Pharmacological therapy was most frequently reported (n=75, 79.8%), followed by physiotherapy (n=70, 74.5%) and orthopedic assistive devices (n=53, 56.4%; Table 4).

Treatment options were reported in a balanced way in 14 HIMs, while 52 HIMs focused on surgical options and 28 on conservative options. The focus varied depending on the type of provider (Table 5).

**Table 3:** Analysis of the content of health information material (HIM) reporting total knee arthroplasty as a treatment option (n=78).

Content				Values, n (%)
**Complications (multiple selections possible)**				
	No mention of any complications in the HIM		28 (35.9)	
	Deep periprosthetic joint infection		39 (50)	
	Implant loosening		30 (38.5)	
	Revision		25 (32.1)	
	Thromboembolic disease		23 (29.5)	
	Other and nonspecific complications^a^		22 (28.2)	
	Stiffness		18 (23.1)	
	Pain (in general)		16 (20.5)	
	Wound complication		13 (16.7)	
	Periprosthetic fracture		13 (16.7)	
	Bleeding		12 (15.4)	
	Instability		10 (12.8)	
	Neural deficit		9 (11.5)	
	Swelling		9 (11.5)	
	Allergy		8 (10.3)	
	Vascular injury		7 (9)	
	Bearing surface wear		6 (7.7)	
	Osteolysis		6 (7.7)	
	Malalignment		5 (6.4)	
	Patellofemoral dislocation		4 (5.1)	
	Extensor mechanism disruption		2 (2.6)	
	Tibiofemoral dislocation		2 (2.6)	
	Blood transfusions		1 (1.3)	
	Mechanical knee noises		1 (1.3)	
	Medial collateral ligament injury		0 (0)	
	Tibial insert dissociation		0 (0)	
**Benefits** **(multiple selections possible)**				
	No mention of any benefits in the HIM		30 (38.5)	
	Pain relief		32 (41)	
	Mobility (general)		24 (30.8)	
	General and nonspecific benefits^b^		20 (25.6)	
	Quality of life		16 (20.5)	
	Functionality in sports		15 (19.2)	
	Functionality in daily activities		10 (12.8)	
	Satisfaction		6 (7.7)	
	Functionality at work		1 (1.3)	
**Anesthetics (multiple selections possible)**				
	Anesthetics not mentioned		57 (73.1)	
	**Types of anesthetics described in HIMs**			
		General anesthetic	19 (24.4)	
		Local anesthetic	16 (20.5)	
		No details on anesthetical procedure	2 (2.6)	
	**Complications with anesthetic** **(multiple selections possible)**			
		No mention of any complications in the HIM	19 (24.4)	
		Nonspecific risks	2 (2.6)	

^a^Complications that are not listed in the standardized list of the Knee Society or are only described unspecifically; for example, scarring (n=7), unspecified signs of inflammation (n=3), problems with soft tissue (n=2), functional limitations (n=2), and surgical errors (n=2).

^b^Benefits that are only described unspecifically; for example, corrections of deformities (n=5), general aspects of quality of life (n=4), symptom reduction (n=4), stability improvement (n=2), and functional improvement (n=2).

**Table 4:** Treatment options for knee osteoarthritis reported in health information materials.

Treatment options (multiple selections possible)				Values, n (%)
Pharmacological therapy (transdermal, oral, injections)				75 (79.8)
**Conservative therapy**				80 (85.1)
	**At least 1 type of physiotherapy mentioned**			70 (74.5)
		Exercise therapy	51 (54.3)	
		Physical therapy	45 (47.9)	
		Not further specified	21 (22.3)	
	Orthopedic assistive devices			53 (56.4)
	Recommendations for lifestyle adjustments			52 (55.3)
	Complementary treatment options			43 (45.7)
	Ergotherapy			4 (4.3)
**Joint-preserving surgical procedures**				60 (63.8)
	Osteotomy			45 (47.9)
	Arthroscopic lavage, debridement			32 (34)
	Arthroscopic cartilage replacement procedures			22 (23.4)
	Arthroscopy (general, without further distinction)			21 (22.3)
	Arthroscopic meniscal surgery			12 (12.8)
**Joint-replacement surgical procedures**				75 (79.8)
	**At least 1 type of TKA^a^** **mentioned**			71 (75.5)
		TKA (general, without further distinction)	38 (40.4)	
		Prosthesis types for TKA	37 (39.4)	
		Fixation methods for TKA	31 (33)	
		Surgical techniques for TKA	26 (27.7)	
	Unicondylar prosthesis			63 (67)
	Patellofemoral prosthesis			17 (18.1)
	Bicompartmental prosthesis			13 (13.8)
	Arthrodesis			1 (1.1)

^a^TKA: total knee arthroplasty.

**Table 5:** Balance of reporting on treatment options by the provider.

Provider	HIMs^a^, n	Presentation of treatment options, n		
		Balanced	Surgery-focused	Conservative treatment–focused
Commercial provider	35	4	15	16
Hospital	26	3	22	1
Medical practice	24	1	14	9
Nonprofit organization	3	2	0	1
Publicly funded provider	3	3	0	0
Professional association	2	1	1	0
Health insurance provider	1	0	0	1

^a^HIM: health information material.

## Discussion

### Principal Results and Implications

This study was designed to investigate the extent to which German-language HIM for the treatment of knee osteoarthritis can facilitate informed choice and to provide an in-depth analysis of contents published in this segment of health information. To this purpose, 94 HIMs from different providers were assessed using the validated MAPPinfo checklist, which operationalizes ethical and evidence-based minimum criteria for HIM aiming to facilitate informed choices. The content of the HIMs was then extracted and analyzed in a standardized manner.

The results indicate a concerning lack of information quality in the HIM analyzed, with an average of only 14.6% (SD 9.4%) of the minimum criteria necessary for informed choices being met. It is noteworthy that none of the HIMs met all the criteria in full (maximum score: 69%).

Deficits in the definition and content criteria are particularly critical. The definition of the target group and the formulation of the objective were fulfilled on average by only 1.1% (SD 10.3%) and 6.4% (SD 20.9%), respectively. Missing or insufficient information in these areas can cause uncertainty among users and contradict the requirements for an informed choice. The description of all possible options and the presentation of the choice between them was fulfilled on average by only 9% (SD 20.6%). Reporting on the natural course of osteoarthritis of the knee was fulfilled on average by only 3.2% (SD 17.6%). Similarly, the appropriate presentation of benefits and harms was fulfilled on average by only 2.1% (SD 14.4%) and 1.1% (SD 10.3%), respectively. It is crucial for patients with knee osteoarthritis to have a comprehensive picture of possible treatments and their consequences so that they can make a decision in line with their preferences and values. These areas were also identified by patients in previous studies as key information needs [11-13,17,18].

We found some differences in information quality between different providers. Health information from publicly funded and nonprofit providers had a higher average information quality of 40.1% (SD 3.6%) and 37.2% (SD 23.1%), respectively. The HIM with the highest quality (69%) also came from a nonprofit organization. On the one hand, this indicates that there is potential, but at the same time, it becomes clear that even these providers do not fulfil all the criteria. Overall, we therefore conclude that the HIM analyzed for the treatment of knee osteoarthritis does not support an informed choice.

According to the providers of the certifications, the certification of HIM is intended to reflect a quality standard. Our analysis showed that HIMs with at least 1 certification have a slightly higher information quality (mean 26.8%, SD 16%) than those without certification (mean 12.7%, SD 5.9%). Due to the predominant focus of these certificates on transparency criteria and the overall low information quality, the quality standard of the certifications appears insufficient. The DNGK certificate stands out here with an average information quality of 40.9% (SD 17.3%), which is mainly due to recommendations from publicly funded and nonprofit providers. However, the content requirements remain considerably below the requirements for an informed choice.

The latest update to the guideline on indication for knee arthroplasty reiterates that nonsurgical treatment should be attempted for at least 3 months before surgery is considered [50]. However, previous studies showed that conservative treatment options are often not sufficiently offered or used [12,13,15,16]. The analysis revealed that 85.1% (80/94) of HIMs mentioned at least 1 conservative treatment option, which at first glance appears to be helpful. However, due to the unbalanced presentation of this information, we have considerable doubts as to whether this really helps to meet information needs. Doctors are highly trusted and are often used as a source of information. [12,23,24]. However, only 3 HIMs from hospitals (n=26) and 1 HIM from medical practices (n=24) presented the treatment options in a balanced way. The majority of these presentations focused on surgical therapies, which could reinforce the impression that conservative options are less relevant and thus strengthen the impression of TKA as the only meaningful option [10,12,14,15]. Decision-making in the treatment of knee osteoarthritis is often complex due to the chronic and fluctuating nature of the condition. Patients are faced with the challenge of addressing their impairments or adapting their lives accordingly while weighing up the benefits and risks of the available treatment options. Their need for information may vary depending on the stage of the disease and their personal circumstances. [10] This emphasizes the importance of comprehensive, balanced, and timely information to ensure that no treatment option is overlooked.

In the 78 HIMs that mentioned TKA as a treatment option, one-third did not mention complications or benefits at all. Serious complications, including but not limited to infections, implant loosening, revision surgery, and thromboembolic events, were mentioned in only up to half of the HIMs.

It is well-documented that patients frequently seek additional sources of information when they are provided with insufficient details by their health care professionals [20]. However, these findings suggest that, due to the generally poor quality of available HIM, this additional search is likely to be unsuccessful and may even place an additional burden on health care systems as doctors have to address the possibly incorrect information given to patients.

Previous studies evaluating the quality of health information on knee osteoarthritis and TKA did not use assessment tools that fully meet the criteria of the Guideline EBHI [29], which defines the standards needed to support informed decision-making, nor have they been extensively validated. Consequently, a direct comparison with our results is not possible. Nevertheless, these previous studies have also identified substantial weaknesses in the HIM they examined [30-36].

The literature repeatedly highlights the issue of inadequate health literacy and the associated difficulties in accessing, interpreting, and using health information effectively to make informed decisions [51]. While we acknowledge and strongly support the need to improve health literacy, it remains essential to ensure that appropriate HIM is available to support informed decisions when adequate health literacy exists. The findings of this study are in concordance with those of previous analyses of HIM, which indicate that such HIMs are currently not available [52,53].

The findings of this study underline the urgent need for health information providers to improve the quality of available HIM. The criteria required to support informed choices should guide these improvements. To address these challenges, initiatives are needed to strategically allocate public resources to the production of high-quality HIM and to encourage greater collaboration between developers of HIM.

The current range of certifications, which are designed to guarantee the quality of HIM, do not meet this objective adequately with regard to the HIM analyzed for the treatment of knee osteoarthritis. Furthermore, these certifications do not equip users adequately with the ability to recognize the individual strengths and weaknesses of HIM. One potential solution might be to establish a certification system based on the MAPPinfo checklist, explicitly assessing criteria necessary for informed choices. Moreover, the incorporation of visual representations of information quality could facilitate transparency for users by clearly indicating which criteria are fulfilled and where deficiencies exist. While numerous tools already exist to help users easily assess the quality of health information, a graphical representation of MAPPinfo information quality could offer a straightforward approach. The findings of our study suggest that patients with knee osteoarthritis seeking treatment-related information should prioritize HIM from publicly funded or nonprofit providers.

### Strengths and Limitations

The strengths of this study include a systematic search strategy, use of the validated MAPPinfo checklist, excellent interrater reliability, and rigorous, systematic documentation of all assessment steps. However, it is important to acknowledge the limitations of the study. First, the search was conducted exclusively using Google, which may introduce a selection bias in an unknown direction. Furthermore, even though the sample was extensive, not all available HIMs could be identified. Second, it could be argued that evaluating HIM against criteria for informed choice may not reflect the intentions of all information providers, as some may claim that they do not explicitly aim to support decision-making. This approach was justified on the basis that users typically cannot discern the intended purpose of a given health resource or determine what information is necessary to support an informed choice. Consequently, it is reasonable to assume that users generally perceive HIM as suitable for decision-making unless it is explicitly and clearly stated otherwise by the provider. A further limitation concerns the temporary nature of the criteria used. At the time of this study, the criteria used in the MAPPinfo checklist represented ethical and evidence-based minimum standards. However, it is conceivable that additional criteria could influence informed choice to varying degrees, although such criteria have not yet been sufficiently validated and therefore could not be assessed.

### Conclusions

Overall, our findings highlight the urgent need to substantially improve the quality of German-language HIM on the treatment of knee osteoarthritis to support patients in making informed choices. The identified deficiencies are fundamental and affect all dimensions of information quality. Furthermore, a substantial proportion of the HIM reviewed does not address critical issues, such as complications and benefits associated with TKA, and only a few present treatment options in a balanced way. Such deficiencies may potentially result in a biased perception of available therapies, which could influence patient decision-making.

Moreover, of the HIM analyzed for the treatment of knee osteoarthritis, only 1 out of 3 available certifications exhibited substantially higher information quality. However, it did not meet the comprehensive requirements needed to support informed choice. The MAPPinfo checklist has the potential to serve as a valuable instrument for more differentiated certification, highlighting both the strengths and weaknesses of HIMs. Furthermore, the establishment of initiatives aimed at enhancing cooperation among providers and strategically aligning public resources should facilitate the development of HIM that meets all the criteria of the Guideline EBHI. Until such initiatives are implemented, patients seeking the highest quality information are advised to consult health information provided by publicly funded or nonprofit organizations.

## Abbreviations

Afgis: Health Information System Action Forum [Aktionsforum Gesundheitsinformationssystem]
DNGK: German Health Literacy Network [Deutsches Netzwerk Gesundheitskompetenz]
Guideline EBHI: Guideline Evidence-Based Health Information
HIM: health information material
MAPPinfo: Mapping the Quality of Health Information
STROBE: Strengthening the Reporting of Observational Studies in Epidemiology
TKA: total knee arthroplasty

## Data Availability

Data on information quality, assessed using the MAPPinfo checklist, is available on Zenodo [54]. Further data is available upon request.
